# Exploring professionals' practices and perspectives on supporting parents with intellectual disabilities: a qualitative study

**DOI:** 10.3389/fresc.2023.1153570

**Published:** 2023-05-30

**Authors:** Romina Rinaldi, Manon Legierski, Erika Wauthia, Emma Mazza, Elise Batselé

**Affiliations:** ^1^ Department of Clinical Orthopedagogy, University of Mons, Mons, Belgium; ^2^ Association pour l'Innovation en Orthopédagogie, Mons, Belgium

**Keywords:** intellectual disabilities, parents, support, professional practices, qualitative study

## Abstract

**Introduction:**

Evidence suggests that parents with intellectual disabilities require appropriate parenting support. However, professional practices vary widely, and several barriers and challenges persist in supporting parents with intellectual disabilities. To identify effective and collaborative practices, this study investigated practices reported by professionals and their roles in providing services to parents with intellectual disabilities.

**Methods:**

Semi-structured interviews were conducted with 22 professionals from three sectors (disability, early childhood, and healthcare), and the content was analysed using inductive thematic analysis.

**Results:**

Thematic analyses yielded four main themes: (1) Perceived professional practices, (2) professional stances, (3) the frame of reference and the ethics of support, (4) the experience of providing support. They are described in terms of content and distribution across sectors to provide an overview of practices as well as potential discrepancies.

**Conclusions:**

This study concludes by developing recommendations on good practices for support professionals to respond as adequately as possible to meet the needs of parents and future parents with intellectual disabilities, which include structural support and guidelines for professionals to provide sensitive, family-centred, and enabling support.

## 1. Introduction

Attention to parents with disabilities, including those with intellectual disabilities, is not new. Throughout the 20th century, the most infamous issue was perhaps the eugenic projects across Europe aimed at the “freely chosen” sterilisation of the “feeble minded” (1). With the growth of disability rights movements, this perspective has changed, and the United Nations Convention on the Rights of Persons with Disabilities mandates that “*State Parties shall take effective and appropriate measures to eliminate discrimination against persons with disabilities in all matters relating to marriage, family, parenthood and relationships* (*…*). (2). This recognition has led to a shift towards a sociocultural perspective on parenting by people with intellectual disabilities placing more emphasis on the *contextual models of parenting* and less on the *capacity* of individuals. These models imply that parenting experience involves not only individual but also environmental risk factors (e.g., socioeconomic disadvantages, lack of social support, co-occurrence of mental health problems) that can be targeted through sensitive support (3–5).

However, *parenting capacity* and *parenting assessment* remain central to the experiences of parents with intellectual disabilities though they continue to be one of the most overrepresented groups in the child welfare system. They face a higher rate of permanently losing custody of their children than other groups of parents and experience unfair procedures (e.g., child welfare cases that remain open for a longer period of time without providing services to improve parenting skills), which are often based on faulty assumptions about the causal relationship between a lower intelligence quotient and the risk of child neglect (6–11). Accordingly, policymakers and the scientific community have been eager to develop and encourage the provision of community-based services for parents with intellectual disabilities that focus on their *support needs* in parenting. Responses to these needs have been considered at several levels, ranging from educational programs or psychoeducational groups to specialised prevention services (12–15).

Consequently, beyond the question of whether people with intellectual disabilities are *capable of* parenting, the issue of how their diverse parenting needs can be recognised and met remains: from early care to the implementation of behavioural boundaries and from fostering their children's learning and developmental opportunities to manage their safety. Parents should be able to exercise these skills while advocating for their role in education and educational decision-making. However, how best to achieve this is a complex issue as professionals must build a trusting and sensitive relationship with these parents (and the family system) to accurately assess and address their support needs in a way that respects their social role, self-determination, and risk management without judicialising family situations.

In an article based on a survey and interviews with professionals, MacIntyre et al. (16) highlighted the high level of variation in professional practice and several persistent barriers and challenges in supporting parents with intellectual disabilities (from identifying parents in need of support but with no clear diagnosis to unplanned/crisis interventions and the reluctance of mainstream services to collaborate). Specifically, they emphasise the importance of contextualising analysis and debate within the social, political, and economic contexts that influence current practices.

To this day however, literature on the experiences of professionals remains scarce and we know little about how they perceive their missions towards parents with intellectual disabilities. An in-depth understanding of their representation and reflections upon these missions may bring insight on potential explanatory variables of barriers and challenges reported by MacIntyre et al. (16). To that end, this study examined professionals' reported practices and the representations of their roles in providing services to parents with intellectual disabilities through a qualitative design and a cross-sectorial perspective.

## 2. Materials and methods

### 2.1. Procedure

This research was conducted as part of the project “Improving the coherence of support for parents with intellectual disabilities in the Wallonia-Brussels Federation”, which was funded by the Office de la Naissance et de l’Enfance (ONE). Data were collected between March and May 2022 in the territory of the Wallonia-Brussels Federation (the French-speaking part of Belgium). This project was approved by the faculty ethics committee. All participants signed an informed consent form before participating in the research.

### 2.2. Participants

Two female researchers (AF and ML) acted as investigators and interviewed an opportunity sample of 22 professionals supporting parents with intellectual disabilities (see Table 1). The investigators had training in either clinical psychology or clinical orthopaedics and were involved in the research project team. The professionals were first contacted via e-mail through the project partners’ network. This e-mail contained an information letter describing the project objectives, the study procedure, what was expected of participants (e.g., planning a meeting to discuss their current practices following a semi-structured interview), and ethical aspects, which was part of the ethical committee submission. They also received a presentation brochure with a video explaining the project details. In the second phase, service providers who had expressed interest in the study were contacted via telephone. They came from various sectors, were active throughout French-speaking Belgium, and had no relationship with the investigators prior to study commitment.

**Table 1:** Characteristics of the sample.

Sector	Services employing participants	Initial training of participants	Functions of participants or services and number of participants involved	Type of support^1^
Early childhood	Office of Birth and Childhood (ONE)	PEPs (*Partenaires Parents Enfants or Parents Children Partners)* can an initial training of social workers, midwives, or community health nurses	PEPs of maternity liaison service- professionals who meet the parents in hospitals one or two days after the child's birth then at parents’ home for the medical and social follow-up on demand (*n* = 3) PEPs working exclusively in prenatal consultations provided within outpatient clinics (*n* = 2) PEPs providing children's consultation at parents’ home or in outpatient clinics (*n* = 3)	Optional
	Office of Birth and Childhood (ONE)	Social worker	*Referrer for abuse* offers an intensification of existing support provided by “front-line professionals” (e.g., PEPs) to help them regarding a complex situation (e.g., risk of abuse) without intervening directly with the family (*n* = 1)	Optional
	Perinatal support service	Midwife and psychologist	Perinatal support services follow families at home in a multidisciplinary manner until a “post-partum relay” can be set up and up to the child is 3 years old. The difference with PEPs is that this service intervenes specifically with mothers with identified social, medical, and psychological difficulties (whereas PEPs intervene to provide support regardless of the mother's situation) (*n* = 2)	Optional
	Maternity home	Specialised educator	Specialised educator who provides individualised support for mothers and their children either in a residential service that offers a time-limited continuous accommodation and social support; or through a post-housing service (outreaching at mother's home) (*n* = 1)	Mandatory
	Intensive family intervention service (IFIS)	Specialised educators	Specialised educators who provide intensive assistance to families in all aspects of daily life of children from 0 to 6 years of age when their medical, social and psychological needs have been identified as compromised by parents (*n* = 2)	Mandatory
Healthcare	Hospitals	Midwives (2) and paediatrician (1)	Midwives and paediatrician provide medical care for the mother and children delivered directly and exclusively at the hospital (*n* = 3)	Optional
Disability	Association for the defence of disability rights/self-representants	Specialised educator	Specialised educator acts as a resource person in a non-profit organisation which supports people with disabilities for self-advocacy (*n* = 1)	Optional
	Disability support services	Specialised educators, social workers	Specialised educators provide support of daily living for people with disabilities, mostly those living outside group homes (*n* = 3)	Optional

Services are provided on an “optional” basis, i.e., only if parents agree to receive them; or on a mandatory’ basis, i.e., as part of a support framework set up by, for example, youth welfare.

Sampling was performed to include the main stakeholders of parenting support to adults with intellectual disabilities. To this end, the sampling considered information related to the professional network collected in the semi-structured interviews. “Early childhood” was the first sector included in the analysis. The research primarily included “child–parent partners” (*Partenaires Enfant Parent-PEPs*) who are the professionals acting on the front lines of parenting support. In the interviews, the professionals were asked to identify the professional actors and services they prioritised in this support mission. The sample was drawn based on this mapping of services; that is, we tried to include the sectors, services, and actors that fulfilled this support mission based on the information collected from the participants.

Based on the collaborations mentioned by the first set of respondents, we attempted to cover all types of services directly involved in supporting parents with intellectual disabilities. In this regard, we distinguish them in terms of *sector.* The *disability sector* supports persons with intellectual disabilities on a continuous basis, most often before they become parents. The *early childhood support sector* responds directly to the presence of one or more children, regardless of whether the parent has intellectual disabilities. Finally, the *healthcare sector* was considered a separate entity. Consequently, each sector operates within a specific mandate. Theoretical saturation of the qualitative data was verified using thematic analysis.

### 2.3. Tools

The semi-structure interviews (see Appendix 1) addressed themes such as the representations of parents and future parents with intellectual disabilities, professional practices, interactions with network partners, the institutional framework in which each professional works, and how this framework influences their intervention methods. The interview structure was pilot tested with an educator from a support service, and the responses were not included in the thematic analysis. Regarding sanitary dispositions related to the COVID 19 pandemic, the professionals were interviewed via video conferencing. The semi-structured interviews lasted about one and a half hours and were recorded with the participants' consent.

Thematic analysis was used to identify the relevant themes in the responses. In line with Braun and Clarke's (17) methodological recommendations, the interview responses (verbatim) were read several times by one member of the research team (RR) to generate initial codes and thematic structures through an inductive procedure; that is, whenever themes were evoked from a data-driven perspective, without trying to fit into an existing coding framework. Independent coding was performed by a second member of the research team (EW), and any discrepancies were resolved through discussion until a full agreement was reached. The participants did not provide feedback on the findings.

## 3. Results

**Figure 1:**
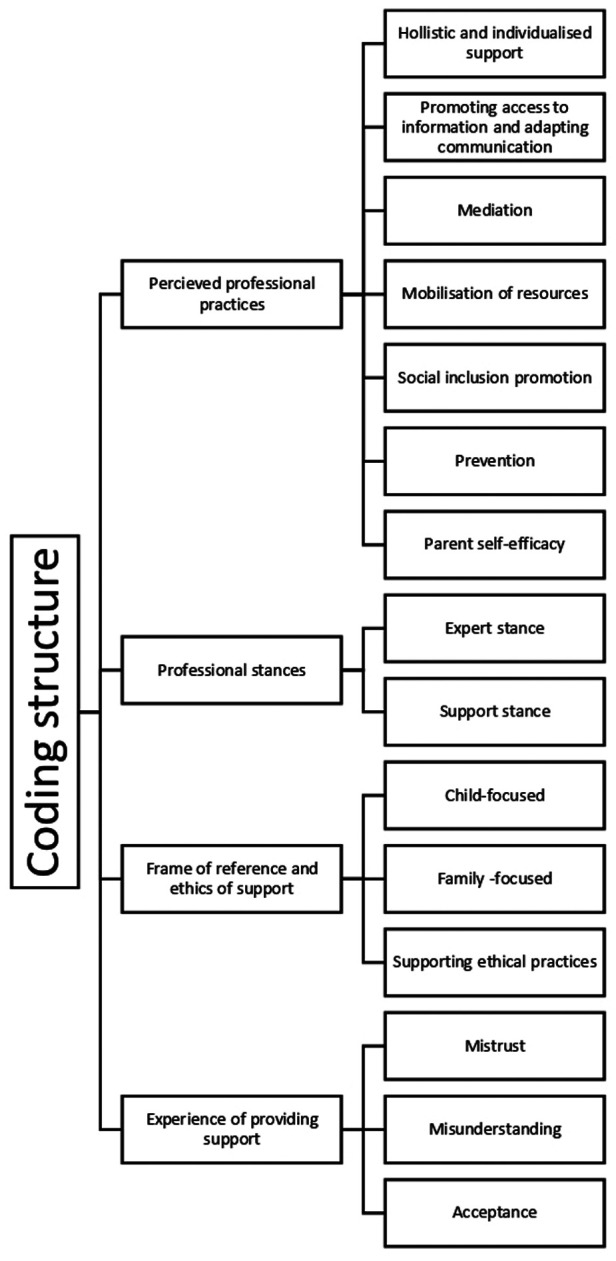
Flowchart of coding structure.

Figure 1 illustrates the main themes and subthemes derived from this analysis, all of which were presented from a content, as well as a sector perspective in Table 2, to analyse potential discrepancies within a theme or subtheme.

**Table 2:** Repartition of sectors within subthemes.

Theme	Subtheme	Early childhood (*n* = 14 participants)	Disability (*n* = 5 participants)	Healthcare (*n* = 3 participants)
		***Number of professionals reporting at least one unit of meaning within the subtheme***		
Perceived professional practices	Holistic and individualised support	14	5	1
	Access to information and adapting communication	13	2	2
	Mediation	11	5	2
	Mobilisation of resources	12	5	2
	Promoting social inclusion	6	4	0
	Prevention	7	5	2
	Promoting parent self-efficacy	6	3	0
Professional stance	Expert stance	9	2	3
	Support stance	12	3	2
Frame of reference and ethics of support	Child-focused	9	3	2
	Family-focused	9	5	1
	Supporting ethics of practices	13	4	2
Experience of providing support	Mistrust	13	5	2
	Misunderstanding	9	2	2
	Acceptance	9	4	3

Thematic analysis revealed four main themes:

(1) Perceived professional practices
(2) Professional stances
(3) The frame of reference and the ethics of support
(4) The experience of providing support

Subthemes will be presented with the number of participants whose discourse was coded within a theme or subtheme (number, *n* =) as well as the number of references (unit of meaning in a subtheme) it involved.

### 3.1. Perceived professional practices

This main theme included responses regarding what professionals considered their missions while supporting parents with intellectual disabilities. First of all, many professionals mentioned the importance of offering **holistic and individualised support** (*n* = 20, 62 references), recognising that the parents' needs varied greatly from one situation to another and that these needs could sometimes go beyond what is considered to be *strictly* related to childcare (e.g., parents needed help with administrative procedures, making medical appointments, help with meals, or budgeting), with varying degrees of tolerance from the professionals on the width and flexibility of their missions

*“I can see they didn't know me at all; they had already run away from me a few times […] they came anyway, they stayed, and I listened to them for two hours, and they told me it was great. I think that listening is a mandatory step to be able to work…the work will come later*”. (PEPs-ONE- Early childhood sector)

*“We know it's hard to work the same way with everyone, yet…My supervisor always says: ‘We do everything but let's not do anything’”*. (Educator-Disability support service-Disability sector)

“*We are social workers. We show empathy, but it might imply to cross limits. We take parents in our car to manage some appointments…think about what it could mean for our insurance!”* (PEPs-ONE-Early childhood sector)

This subtheme was evoked in each sector (see Table 2). One healthcare professional mentioned this mission with concerns that professional partners and parents would not understand her genuine mission if she provided this form of holistic support. Moreover, this concern is not limited to the healthcare sector. In both the disability and early childhood sectors, some professionals fear that this holistic work will not allow for personal and professional boundaries or that it sometimes went beyond their official missions, what their hierarchy would tolerate, or even what was acceptable regarding the legal aspects of work (e.g., insurance). Others considered that accepting work outside the strict boundaries of childcare and education was a way of creating an alliance with parents and making them more available for collaboration. Supporting them in various dimensions was considered a way of recognizing their needs and therefore establishing a climate of trust.

Professionals' discourses hence thoroughly highlighted a form of flexibility and creativity in practices, particularly concerning another subtheme: **promoting access to information and adapting communication channels to parents' needs** (*n* = 17, 62 references). This mission was highlighted across sectors, with the highest representation in the early childhood and healthcare sectors. One participant in the disability sector mentioned that her service created a whole set of visual aids for parents with intellectual disabilities, which was shared with other professionals in their network, specifically within the early childhood sector.

*“So how do I show the brochures if the parents cannot read? For the understanding of milk measures and bottles? We work with graphs, with signs that we put on the bottles or with small tools that we create”*. (Referrer for abuse—ONE- Early childhood sector)

“*The child needs 12 drops of vitamin. Well, how were we going to explain it? We made little drawings and so we drew 12 drops, we drew the bottles… we stuck it in the child's health book*”. (Midwife- Healthcare sector)

Visual and practical tools (e.g., visual marks, pictograms, drawings, timelines) were often mentioned by professionals, accompanied with a warning that these tools may help to some extent but tend to become insufficient when it comes to the more complex needs of children, such as being stimulated by playing and learning, having behavioural boundaries, or having their emotional needs recognised. Professionals suggested that these needs may be better met through informal support (e.g., family support) or the inclusion of children in nurseries and childcare.

Regarding communication channels, professionals emphasised the importance of direct (e.g., showing instead of explaining, being present) and flexible contact (e.g., allowing for various ways of communicating, such as visiting, using messaging, or videoconferencing).

*“When there's a problem, she knows she has my phone number, she's already called me and said: ‘I think he has a cold.’ With another mother, I would have asked questions on the phone, and I would have given advice. With her, I had her come in right away, so I could explain things to her face-to-face and show her child”*. (Paediatrician-Hospital- Healthcare sector)

*“I went with her to the maternity ward. We gave her a tour of the delivery rooms; we explained the epidural and everything in terms that are more suitable for her”.* (PEPs-ONE- Early childhood sector)

This adaptation of information and communication channels was sometimes used for **mediation** purposes (*n* = 18, 48 references), which was identified as another subtheme. It refers to the role played by some professionals, specifically by how they made themselves available for the health follow-up of the mothers to ensure that the information was given to them in an adequate format and clearly understood. However, mediation—defined here as acting in-between—includes broader examples than communication with healthcare. Professionals reported that they often facilitate communication and procedures with various services, such as youth protection and other parental support services, as well as childcare and school. This mediation role seems to be fostered by a trustful and/or long-term (continuous) relationship between parents and professionals and is also evoked as a way to ensure continuity in support (i.e., bridging the gap from one service to another). This mission was highlighted in all sectors. Specifically, each sector mentioned acting as mediators with one or the two other sectors involved in this study. Professionals from the early childhood and disability sectors also evoked contact and collaboration with social services, administrative services, housing services, schools, and child protection services whenever they were involved. Therefore, this mission was evoked in a narrower sense by healthcare professionals who mostly reported working with early childhood services.

Another mission mentioned was the **mobilisation of the resources** needed to ensure the exercise of parenthood in the best possible conditions (*n* = 19, 56 references). Such resources encompassed housing, finance, and access to services (e.g., childcare, nursery) but also included the management of transitions from one service to another when temporary mandates were exhausted. In some cases, it may also consist of a decision to appeal to judicial child protection services to move from freely accepted support to mandatory support. This mission seems transversal as it was represented in each sector. In contrast to the mediation subtheme, healthcare professionals reported working with child protection services. The disability and early childhood sectors extended the network involved in this subtheme compared to the mediation theme, adding daycare, transportation, cleaning services, job search, psychological counselling, and many more to the extent of this mission.

Some professionals also mentioned **promoting social inclusion** (*n* = 10, 14 references) of parents and children as part of their mission, mainly in the form of informal support fostering or promoting community experiences.

*“As a team, we thought about how to expand the network and how to find front-line partners, neighbours, and family members who could intervene more easily”.* (Educator-Disability Support Service-Disability sector)

These experiences were sometimes mentioned as a way of broadening learning opportunities and creating a variety of environments that can stimulate and nurture children.

*“Today, there is an egg hunt for the kids. It allows us to set up more community activities […] It also allows us to be in a different approach…maybe less in a meeting where… It's hard to be in something authentic […] We realize that living with the people has sometimes more impact than being in a professional relationship”.* (Psychologist-Perinatal Support Service- Early childhood sector)

**Prevention** (*n* = 14, 29 references) was also mentioned as a mission field and was highlighted across sectors. It mostly focuses on the idea of planning for parenthood, which, on the one hand, could take the form of reflection with parents on the reception of the unborn child, information on various services that could support them, and on the other hand, birth control and information on contraception. Birth control was mostly discussed regarding medical aspects. Some professionals of the disability sector evoked it in the context of helping adults with intellectual disabilities consider parenthood in a broader life project and to reflect on the status of adults beyond the role of parents.

“*Sometimes disabled people don't have a job: that's already a missed step! They are not married: that's another missed step! It's a bit of a cliché. We must deconstruct this representation that a child is a goal in life. Maybe some other things in their life should be valued*” (Educator-Disability Support Service- Disability sector).

The position of professionals in the early childhood sector was more complex, with health-related prevention (e.g., birth control, assessing children's development) and discussions on parenthood projects. These discussions about parenthood were repeatedly evoked with the theme of “reparations” (i.e., whenever mothers with ID wanted to be pregnant again after a child was removed from home).

Finally, some professionals explicitly mentioned that their mission involved establishing or restoring confidence in parenting skills and **promoting parent self-efficacy** (*n* = 9, 20 references). As a result, they saw their mandate as promoting parental *empowerment* and a sense of self-efficacy. This subtheme was also found in the early childhood sector and was most represented in the disability sector.

*“We trusted them, and it went well. When you invest in them positively, they are parents who also manage to develop skills… They need… I keep telling the mother: ‘I'm proud of you; you're doing well*’” (PEPs-ONE- Early childhood sector)

*“We really try to emphasise parenting skills. To tell them that it's a virtuous circle: the more parenting skills they have, the more it reflects in the development of the child, and the more it goes back to their parenting skills”.* (Educator- Disability Support service- Disability sector)

### 3.2. Professional stance

In professional discourse, missions were carried out through *stances* described as **supporting stances** (doing together, being present for the parent, promoting parental expertise, fostering parental competence) (*n* = 17, 49 references) or **expert stances** (*n* = 14, 30 references). These expert stances vary on a continuum ranging from “*doing things in the parent's place”* and assisting with decision-making to “*assessing the parent”* and parental competence. While this discourse could sometimes evoke a position of authority in which the professional imposes on the parents what they perceive to be good or bad for the child, on other occasions, some stressed the importance of giving advice without necessarily issuing injunctions.

The results highlight that many professionals assume both types of stances (see Table 2), although proportionally, the disability sector is less represented in both stances. Sometimes, expertise is part of the professional's mandate, but they will feel the complexity of the situation and the difficulty of collaborating with the parent and will describe a more supportive stance. This was described especially in relation to situations in which people with intellectual disabilities have been placed in a position of “chronic dependence” on services or when they themselves have experienced placements. One stance, therefore, does not “cancel out the other”. Professionals' discourse also allows us to understand that their expertise stance is not always a personal claim. Sometimes it is the families of parents with intellectual disabilities who confer it, or other professionals who put the professional in this role when it is not their mandate or way of working. However, professionals may not be comfortable with this stance.

In direct relation to these attitudes, the professionals mentioned that some parents might feel dependent on services during parenthood or may even be deprived of their ability to make appropriate decisions.

*“These are people who are always ringing us up because they don't dare to do anything without our input”.* (PEPs-ONE- Early childhood sector)

### 3.3. The frame of reference and the ethics of support

Missions were reported to be influenced, for the most part, by the need **to focus on the child** and consider his or her best interests (*n* = 14, 28 references). Indeed, professionals seem to be organised unanimously around this dimension. However, this primary frame of reference also gives rise to an ethical support dimension. Several participants mentioned a form of conflict between their primary focus on the child and the desire **to focus on the family** (*n* = 15, 23 references). Should one take precedence over the other? The professionals felt that positioning was often required in practice.

“*They are focused on the child's needs, it makes sense. But if you don’t take care of the mother too, you’ll get nothing! How could she manage to take care of her kid if she's not well herself?”* (Educator- Maternity Home- Early childhood sector)

To resolve this perceived “dilemma”, some professionals reported having set personal guidelines, whereas others referred to the transversal orientation of the network or service in which they worked. However, it must be noted that ethical reflection was less acute when the professional was faced with situations in which previous placement decisions had already been made. In this case, they considered that decision-making should not rely on ethical reflexion but also seek to minimise risk-taking and focus exclusively on the child. Child- and family-focused frames of reference were mentioned across sectors, although family-focused frames seemed less represented among healthcare participants and more represented in the disability sector.

*“I find it very interesting and important that people with disabilities have the same rights as everyone else. But sometimes it's very complicated. We see that in the end, these are children who end up being removed from their family […] And then, each person is different, each situation is unique. […] I don't think there are any good answers on this subject”.* (Coordinator-Perinatal Support Service- Early childhood sector)

To support **ethical practices**, professionals also mentioned relying on mechanisms such as transparency in exchanges with parents (e.g., if a report is due, parents are involved in its preparation and informed of the previous and subsequent stages), questioning one's own representations of what parenthood should be, and considering one's role as a professional while providing support.

*“We know that the child doesn't receive too much investment, but between not receiving much and receiving nothing […]I tell myself that we are not here to save people”* (Coordinator- Intensive Family Intervention Service- Early childhood sector)

*“We never have meetings without parents. At the very least, if we have something to say, we give each other a call. But we try to remain transparent with the parents.”* (Educator- Disability Support service: Disability sector)

For healthcare participants however, ethical support was related more directly to questioning the decision-making process about unconsented birth control and risk management (e.g., how professionals should react when they are alerted by parents' behaviour after birth).

*“Professionals will sometimes impose a contraception after the birth as soon as the mother returns home because they are afraid that she will get pregnant again and they know that parents are not capable of managing their child. I often tell myself: ‘Is it ethically right to do that?’”* (Midwife- Healthcare sector)

### 3.4. Experience of providing support

Professionals had the opportunity to express their views on how parents with intellectual disabilities perceived the support services offered. From a sectorial perspective (see Table 2), it appears that providing support to parents with ID is challenging for most professionals.

One of the salient elements of the discourse hence referred to **the mistrust** (*n* = 20, 52 references) felt by professionals regarding their intervention which may be present from the moment the desire for a child was expressed. Parents’ main concern is the threat of not being considered up to the task and, as a result, being subjected to a procedure for removal or placement of the child already born or to be born. This mistrust stems from a lack of understanding of the role of professionals or may sometimes be part of a life path marked by this type of breakdown. This fear tends to make parents shy away from or avoid services because they have the impression that their sole purpose is to monitor and evaluate their parenting skills.

However, in the disability sector, mistrust was mostly evoked regarding other services involved, including early childhood support, and sometimes regarding the relationship of professionals with this sector. Mistrust may therefore emerge from this collaboration and not from their presence itself with parents; notably, the professionals often knew them before they become parents. In contrast, mistrust was more directly oriented toward professionals in the early childhood and healthcare sectors.

*“The difficulty came from this fear of placement. The father claimed that he was there for his baby. […] We had to work on getting past that and that it was not bad to need support. It's okay to ask for help; the goal is to be able to meet the baby's needs as best as possible. It was necessary to trivialise our intervention, to explain to them that there are indeed families who need to be supported”.* (Family worker—Intensive Family Intervention Service- Early childhood sector)

*“When we came to the house, he stayed upstairs in his room; he didn't want to come down. He would say: ‘They think I'm stupid too’. He also often repeats: ‘I'm not disabled (…) They show us how to make a purée, and my wife is happy’. He, instead, didn't see it well because he says, ‘they want to do everything for us.’”* (PEPs- ONE- Early childhood sector)

Professionals also mentioned **acceptance** of support in some situations (*n* = 16, 40 references), with various factors elicited as promoting it. First, the establishment of a trusting relationship and mutual commitment. To build and maintain this relationship, transparency regarding missions and mandates was evoked as a key dimension of practices.

Other acceptance factors elicited by professionals include adapting to the needs of the whole family, recognition of people in their role as parents, and continuity in the support of parenthood (e.g., when professionals have been able to work with parents at different times in their lives and in different contexts and environments, they have been able to build a strong partnership).

*“I said to the parents: ‘It's good enough that you're worried about your child's health, there are parents who don't care about that’. The least I could do was to bounce on it and congratulate them on their responsibility. One step after another, I managed to get in the house. From then on, things got better and better”.* (PEP-ONE- Early childhood sector)

Finally, professionals also reported **misunderstanding** from parents (*n* = 13, 21 references) regarding their intervention without associated mistrust, *especially* when many people were involved in the same situation. Here again, relationships and communication were mentioned as fundamental processes for overcoming these difficulties.

*“Sometimes they get lost; they don’t find their way around. In our service alone, there is already a difference between the shrink and the midwife. Some people don’t know the difference. I think it takes time; it takes time to get to know each other. You must live with them*”. (Psychologist-Perinatal Support Service- Early childhood sector)

## 4. Discussion

This study sought to examine the practices reported by professionals as well as the representation of their roles in providing services to parents with intellectual disabilities. A total of 22 professionals supporting parents with intellectual disabilities participated in semi-structured interviews addressing various themes. The analyses confirmed the diversity of the missions within this target group, and the fact that missions seem to be defined by the family's needs rather than the strict issue of childcare and education. This element, presented as a good practice within the scientific literature (18–21), can, however, be difficult for some professionals who feel that they are overstepping their boundaries and mandates if they switch their actions from supporting the parent–child relationship to supporting the family system. This issue was mostly salient in the *early childhood* sector, for which professionals reported such practices. However, there were also many concerns regarding institutional, legal, or professional boundaries. Therefore, professionals should be able to receive diverse structural support in their missions, including structural measures allowing flexibility in their mandates and work environments. Indeed, family-centred support, beyond the framework of learning parenting skills, seems to be a condition for maximising the usefulness of support from the parents' perspective, thus ensuring the continuity of the interventions and a relationship of trust with the (future) parents. Without perceived usefulness and collaboration, the possibility of supporting parents is compromised.

Professionals reported parents often mistrusted or misunderstood their work. This mistrust is fuelled by the fact that parents perceive the professional's presence as a challenge to, or denial of, their skills, or even their status as adults and parents, or by the fear that professionals may seek to play a “supervisory” role and initiate or precipitate placement measures. Misunderstandings are encouraged by the diversity of actors, services, and information in these situations. It should be noted that while these experiences were reported by professionals in each sector, the disability sector evoked mistrust regarding other professionals (e.g., those in the early childhood sector), as well as regarding collaborations they might have with these professionals. On the other hand, because professionals from the disability sector often collaborate with people with intellectual disabilities prior to parenthood, they have the opportunity to develop mutual knowledge and build a trusting relationship in which these people are primarily considered *adults* with needs, rights, and duties. Their experience of support, although not completely free of challenges, can be considered more favourable in terms of relational context and temporality; thus, supporting the partnership of healthcare and early childhood professionals with those of the disability sector may improve coordinated support.

Another issue is the professional stance. Our results indicated that this stance could be broken down in the reported practices along a continuum ranging from “doing together” (support) to “doing instead of/deciding for”/ “and assessing (expertise)”. The results of this study showed that professionals often had to jump back and forth between their positions of authority and guidance. These developments, which corroborate those of previous research (16, 22), call into question how the explicit and implicit roles assigned to professionals can influence collaboration and co-construction processes.

Regarding the support stance, it should be noted that the professional discourse did not focus much on the resources to be mobilised by parents with intellectual disabilities, their own expertise, or their parental status. However, the available data suggest the importance of recognising this parental expertise and the fact that there are different types of knowledge and expertise (16). Additionally, recognising parents' strengths and resources and being aware of what they consider important are factors that support adherence to formal and informal support for their parenting (7, 16, 23). Additionally, how can professionals place themselves in an authentic support stance if they do not consider parents' expertise and resources?

Although some professionals evoked social inclusion as a protection factor and promoted it as part of their mission, informal support was rarely mentioned, and when it was, it was described as a favourable context that was already in place; participants had little idea of how they themselves could mobilise this support. As a consequence, professionals consider that they bore the responsibility of providing parents and children with resources and support, while actually lacking time and resources to take such a responsibility. This was particularly salient in the early childhood sector, in which professionals often consider social inclusion as an issue for the children (e.g., children being “stimulated” because they have the opportunity to frequent day care) but considering that the best option for parent would be intensive professional support or coparenting rather than considering that social exclusion may increase their vulnerability when the mandates of such services come to term. Informal support (e.g., help from family, peers, and neighbours) remains crucial in the help-seeking process and can meet the needs of a significant proportion of parents. However, available research suggests that these informal networks, although valued by parents with intellectual disabilities (21, 24, 25), may be rare and difficult to mobilise (17, 26).

Finally, stances and reported practices seemed to be modulated by professionals' frames of reference. Most professionals mentioned considering the child's best interests as the central point of their practices and decision-making processes. The child's best interests refer to the fact that, in the event of divergence between the interests of the child and those of the parents, the former takes precedence over the latter (27). However, in this study, the frame of reference was often considered a broader “focus” of practices and missions. Professionals may have reported that they were primarily *working for the children,* which is a distinct perspective of the child's best interests relying on the idea that the child's needs can be sufficient to guide parental support. This question arises particularly within practices when one considers an ethical knot resulting from the apparent opposition between the fundamental right of persons with intellectual disabilities to be parents, on the one hand, and considering the child's needs on the other. Notably, these dimensions are largely considered mutually exclusive in professional discourse. That said, this theoretical opposition raises questions about the belief system regarding parenting among adults with intellectual disabilities. In this regard, available data suggest that a *validist* vision —one in which professionals demand that parents meet specific standards in the areas of care, feeding, protection, and education—of parenthood remains widespread (28, 29). Consequently, a contradiction is played out between, on the one hand, the desire to see the development of so-called “normal behaviours” in accordance with the norms and values enforced in our society and, on the other hand, the recognition of the person with intellectual disabilities as a person with a disability and with specific needs (30). In this vision, access to parenthood may be. From then on, the child's interests and the parent's rights are considered “polarised”. However, creating antinomy between the childs and parents' rights and needs may reinforce negative perceptions and mistrust of services. Considering that the best interests of the child are a structuring principle for professionals and the political and legal systems that organise them, family-centred support, identification of support needs, flexible responses (e.g., intensification of a system in place in the event of a crisis), and implementation of support that respects parental expertise and status can represent effective preventive measures that make it possible to achieve this consideration for the child's best interests without structurally denying the rights and needs of parents or those of the family.

### 4.1. Limitations

This study has several limitations. The first is the use of an opportunity sample which, although intended to be representative of the service structure, cannot fully reflect all practices. In addition, qualitative methodology is a useful but preliminary approach. Based on the dimensions elicited in this study, a survey approach conducted using stratified sampling should deepen the results and better determine how the representations of support practices are influenced by the mandates and professional cultures of different targeted sectors. Similarly, our data do not allow for a detailed analysis of practices within the network, although interprofessional collaboration and articulation of services are major issues in the deployment of support. Finally, it should be noted that pprofessionals talked about their work with parents with intellectual disabilities in general. Although this study aims to extract cross-sectional information and recommendations, this group should not be considered as homogeneous. The life situations of these parents can vary greatly; particularly in terms of where they live, and this undoubtedly influences the perspective, practices and decision-making of professionals supporting their parenting. A comparative perspective that includes the discourse on parents with intellectual disabilities would also be useful to complete current data and provide more inclusive recommendations.

### 4.2. General conclusion

In conclusion, the results of this study highlight that the reported practices of professionals supporting parents with intellectual disabilities are varied and displayed in a holistic, family-centred, and individualised approach that is influenced by stances, frame of reference, and consideration of the boundaries of the professional's mandate. These results highlight the need to develop recommendations on good practices for support professionals to respond as adequately as possible to meet the needs of parents and future parents with intellectual disabilities and to provide them with high-quality and inclusive support policies. With this in mind, we propose that when an investigation is required, parents should receive information from independent and competent sources that can provide advice and advocacy. We also suggest that, in the event of placement, parents should be given support to maximise their chances of improving their parenting skills. It is common for parents to react intensely to the removal of a child, particularly when a new experience rekindles feelings from the previous experience. Emotional support should be offered to ensure that behaviours and comments do not necessarily jeopardise the chances of the family being reunited. In addition, good practices must include explanations of each stakeholder's roles and maximum continuity in the parent follow-up. Furthermore, the services offered must be clear, accessible, and cohesive for the scope of the service to be understood and the breakdown of support procedures to be minimised. Professionals should receive support for their own practices with a clear yet flexible framework that allows for multi-professional work, as well as flexible, individualised, and family-focused forms of interventions as these factors, including the choice of support's form, have been highlighted as “acceptance factors” that improve both the process and outcomes of the support and lead to overall satisfaction of both professionals and families. Professionals should be able to conceive of their own places in a wider network involving both professionals and informal resources. Therefore, their role in the social inclusion of parents with intellectual disabilities should be emphasised. Finally, professionals should be guided and supported since their missions take place in complex and deeply ethical contexts, particularly through continuous training and team/individual supervision.

## Data Availability

The raw data supporting the conclusions of this article will be made available by the authors upon request.

## Appendix 1 Examples of main questions within the semi-structured interviews

(1) **Ice-breaking questions**
(•) *What is your typical day like?*
(2) **Perceived practices in supporting parents with ID**
(•) *Can you describe your missions with parents with ID?*
(•) *How do you describe your role and missions to parents?*
(•) *Does supporting parents with ID involve specific aspects of your work (compared to other parents)?*
(3) **Resources and obstacles in supporting parents with ID-** *I propose taking a few minutes to think about a situation of parents with ID you supported or are currently supporting.* (This statement is used as a starting point to deepen the roles and missions but also the means and obstacles.)
(•) *What was the initial request with this situation?*
(•) *Were other professionals or services involved?*
(•) *What obstacles did you encounter in this situation? And in your work with parents with ID, to a broader extent?*
(•) *What helped you in this situation? And in your work with parents with ID, to a broader extent?*
(4) **Collaboration**
(•) *Who are your privileged partners for supporting parents with ID?*
(•) *How would you describe your work with these partners?*
(5) **Support needs**
(•) *What would you need to better support parents with ID?*
(•) *What advice would you give to promote the quality of the interventions?*
(6) **Ethical dimension of support**
(•) *What are the ethical aspects of your work with parents with ID?*

## References

[1] Krimbas C. Eugenics in Europe. In: Smelser NJ Baltes PB, editors. International encyclopedia of the social & behavioral sciences. Oxford: Pergamon (2001). p. 4905–12. ISBN 9780080430768. 10.1016/B0-08-043076-7/03397-0

[2] United Nations. Convention on the rights of persons with disabilities (2006).

[3] McGaw S Scully T Pritchard C. Predicting the unpredictable? Identifying high-risk versus low-risk parents with intellectual disabilities. Child Abuse Negl. (2010) 34(9):699–710. 10.1016/j.chiabu.2010.02.006 20674975

[4] Schuengel C Kef S Hodes MW Meppelder M. Parents with intellectual disability. Curr Opin Psychol. (2017) 15:50–4. 10.1016/j.copsyc.2017.02.022 28813268

[5] Wade C Llewellyn G Matthews J. Parent mental health as a mediator of contextual effects on parents with intellectual disabilities and their children. Clin Psychol. (2015) 19(1):28–38. 10.1111/cp.12055

[6] Aunos M Pacheco L. Able or unable: how do professionals determine the parenting capacity of mothers with intellectual disabilities. J Public Child Welf. (2021) 15(3):357–83. 10.1080/15548732.2020.1729923

[7] Collings S Spencer M Dew A Dowse L. She was there if i needed to talk or to try and get my point across: specialist advocacy for parents with intellectual disability in the Australian child protection system. Aust J Hum Rights. (2018) 24(2):162–81. 10.1080/1323238X.2018.1478595

[8] DeZelar S Lightfoot E. Who refers parents with intellectual disabilities to the child welfare system? An analysis of referral sources and substantiation. Child Youth Serv Rev. (2020) 119:105639. 10.1016/j.childyouth.2020.105639

[9] Lightfoot E DeZelar S. The experiences and outcomes of children in foster care who were removed because of a parental disability. Child Youth Serv Rev. (2016) 62:22–8. 10.1016/j.childyouth.2015.11.029

[10] Lightfoot E Slayter E. Disentangling over-representation of parents with disabilities in the child welfare system: exploring child maltreatment risk factors of parents with disabilities. Child Youth Serv Rev. (2014) 47:283–90. 10.1016/j.childyouth.2014.10.001

[11] McConnell D Llewellyn G. Stereotypes, parents with intellectual disability and child protection. J Soc Welf Fam Law. (2002) 24(3):297–317. 10.1080/09649060210161294

[12] Coren E Ramsbotham K Gschwandtner M. Parent training interventions for parents with intellectual disability. Cochrane Database Syst Rev. (2018) 7(7):CD007987. 10.1002/14651858.CD007987.pub3 30004571 PMC6513025

[13] Murphy G Feldman MA. Parents with intellectual disabilities. New York, NY: Wiley (2002).

[14] Tarleton B Turney D. Understanding “successful practice/s” with parents with learning difficulties when there are concerns about child neglect: the contribution of social practice theory. Child Indic Res. (2020) 13(2):387–409. 10.1007/s12187-019-09682-y

[15] Wade C Llewellyn G Matthews J. Review of parent training interventions for parents with intellectual disability. J Appl Res Intellect Disabil. (2008) 21(7):351–66. 10.1002/14651858.CD007987.pub3

[16] MacIntyre G Stewart A McGregor S. The double-edged sword of vulnerability: explaining the persistent challenges for practitioners in supporting parents with intellectual disabilities. J Appl Res Intellect Disabil. (2019) 32(6):1523–34. 10.1111/jar.12647 31318123

[17] Braun V Clarke V. Using thematic analysis in psychology. Qual Res Psychol. (2006) 3:77–101. 10.1191/1478088706qp063oa

[18] Llewellyn G Brigden D. Factors affecting service provision to parents with intellectual disabilities. Child Youth Serv Rev. (1995) 20:97–112. 10.1080/07263869500035481

[19] McConnell D Llewellyn G Bye R. Providing services for parents with intellectual disability: parent needs and service constraints. J Intellect Dev Disabil. (1997) 22(1):5–17. 10.1080/13668259700033251

[20] Meppelder M Hodes M Kef S Schuengel C. Parents with intellectual disabilities seeking professional parenting support: the role of working alliance, stress and informal support. Child Abuse Negl. (2014) 38(9):1478–86. 10.1016/j.chiabu.2014.04.006 24856130

[21] Wilson S McKenzie K Quayle E Murray GC. The postnatal support needs of mothers with an intellectual disability. Midwifery. (2013) 29(6):592–8. 10.1016/j.midw.2012.05.002 23123156

[22] Spencer M Llewellyn G. Working things out together: a collaborative approach to supporting parents with intellectual disabilities. In: Bigby C Fyffe C Ozanne E, editors. Planning and support for people with intellectual disabilities: Issues for case managers and other professionals. London: Jessica Kingsley (2007). p. 71–190.

[23] Strnadová I Collings S Loblinzk J Danker J. Parents with intellectual disabilities and their perspective of peer support: “it depends on how they give it”. J Appl Res Intellect Disabil. (2019) 32(4):879–89. 10.1111/jar.12579 30790398

[24] Wołowicz-Ruszkowska A. How Polish women with disabilities challenge the meaning of motherhood. Psychol Women Q. (2016) 40(1):80–95. 10.1177/0361684315600390

[25] Wołowicz-Ruszkowska A McConnell D. The experience of adult children of mothers with intellectual disability: a qualitative retrospective study from Poland. J Appl Res Intellect Disabil. (2017) 30(3):482–91. 10.1111/jar.12322 28070932

[26] Llewellyn G McConnell D Bye R. Perception of service needs by parents with intellectual disability, their significant others and their service workers. Res Dev Disabil. (1998) 19(3):245–60. 10.1016/S0891-4222(98)00006-7 9653801

[27] David Vainberg L Vardi A Jacoby R. The experiences of parents of children undergoing surgery for congenital heart defects: a holistic model of care. Front Psychol. (2019) 10:2666. 10.3389/fpsyg.2019.02666 31827455 PMC6890854

[28] Bless DO Petitpierre G. Les parents ayant une déficience intellectuelle–Entre autonomie et soutien social indispensable (2017).

[29] Hoghughi M. Parenting: an Introduction. In: Hoghughi M, editors. Handbook of parenting: Theory and research for practice. London: Sage Publications (2004). p. 1–18.

[30] Mercier M Carlier G Lotin B. Liens: Parentalité des personnes déficientes mentales (2004).

